# Traumatic dentoalveolar injury, tooth wear, and periodontal disease in working and non-working dogs (2018–2022)

**DOI:** 10.3389/fvets.2026.1816961

**Published:** 2026-05-07

**Authors:** Shawna Han, Alexander M. Reiter, Darko Stefanovski, Ana C. Castejón-González

**Affiliations:** 1 MSPCA-Angell Animal Medical Center, Dentistry and Oral Surgery Service, Boston, MA, United States; 2 Tierzahnzentrum München, Munich, Germany; 3 Department of Clinical Studies-New Bolton Center, School of Veterinary Medicine, University of Pennsylvania, Kennett Square, PA, United States; 4 Department of Clinical Sciences and Advanced Medicine, School of Veterinary Medicine, University of Pennsylvania, Philadelphia, PA, United States

**Keywords:** periodontal disease, strategic teeth, tooth wear, traumatic dentoalveolar injury, working dog

## Abstract

**Introduction:**

Periodontal disease and tooth fracture are the main reasons for tooth extraction in dogs. The objectives of this study were to compare the prevalence of traumatic dentoalveolar injury (TDI), tooth wear, and periodontal disease in working dogs (WD) and non-working dogs (NWD).

**Methods:**

Medical records of WD and NWD presented during a 4.5-year period were reviewed to identify TDI, tooth wear, and periodontal disease in WD and NWD. Periodontal disease was evaluated only in strategic teeth (canine, maxillary fourth premolar, and mandibular first molar). TDI and tooth wear were evaluated in all teeth.

**Results:**

WD had 6.3 ± 6.4 TDI and 12.7 ± 11.5 teeth with tooth wear per dog. NWD were diagnosed with 1.3 ± 1.7 TDI, and 1.9 ± 4.9 teeth with tooth wear per dog. The risk of having a TDI was 3 times higher in WD than in NWD, and the likelihood of having tooth wear was 66% higher in WD than in NWD. WD had fewer strategic teeth with severe stages of periodontal disease than NWD.

**Discussion:**

TDI, tooth wear, and periodontal disease are frequent in WD, and the prevalence of TDI is higher than previously reported. Oral examinations should be included in routine annual evaluations in WD to accurately identify and treat traumatized teeth and periodontal disease.

## 1 Introduction

Working dogs (WD) are tasked with a specific job, normally outside of companionship (1). It is estimated that there are up to 50,000 WD in the United States (e.g., conservation, search and rescue, explosives, narcotics, food) and are often selected from working lines with strong olfactory capability and strong interest in toys or food (2, 3). Apprehension or patrol dogs are a non-lethal use of force and used to track and subdue suspects with physical apprehension, which may be executed with contact (1). WD should have healthy full dentition with a scissor bite providing the strongest grip (4). Oral pain, dental and periodontal disease, and tooth loss may affect their performance and compromise their working career.

Periodontal disease and tooth fractures are the main reason for tooth extraction in dogs (5). These pathologies can result in oral pain, infection, and osteomyelitis. Breed, size, body weight, and age are risk factors for periodontal disease (6). Traumatic dentoalveolar injury (TDI) affects the tooth (crown/root) and/or the tooth-supporting structures (periodontal ligament/alveolar bone) and are sustained as a direct result of trauma. A previous study showed a prevalence of TDI in dogs and cats to be 26.2% (7). This prevalence increases to 43.6% in military dogs and 72.1% in dogs and cats with maxillofacial trauma (8, 9). A recent study in military dogs evaluated enamel-dentin and enamel-dentin-pulp fractures, but crown, crown-root, or root fractures were not differentiated (8). Tooth wear (abrasion and attrition) is not classified as a TDI, but it is a form of relatively low intensity trauma that can cause tooth sensitivity and pulpitis. Pulp exposure is also possible if the tooth structure is removed faster than the odontoblasts can synthetize dentin (10).

The objective of the present study was to compare the overall prevalence of TDI, tooth wear, and periodontal disease in working (WD) and non-working dogs (NWD). We hypothesized that WD have more TDI and decreased severity of periodontal disease than NWD.

## 2 Materials and methods

### 2.1 Patient selection

Medical records of WD and NWD presented for an anesthetized oral examination to the Matthew J. Ryan Veterinary Hospital of the University of Pennsylvania from May 2018 to October 2022. WD was defined as a dog tasked with a specific job assigned with a law enforcement agency and NWD as a companion dog without a specific job. Dogs were included if they presented for an oral procedure and if they were 18 months of age or older. All WD that fit the inclusion criteria were included in the WD group. A random number generator was used to select the same number of NWD within the same period of time.

Signalment and body weight were recorded. Patients in each group (WD, NWD) were assigned to one of the three weight (<10 kg, 10 to 20 kg, >20 kg) and age (up to 36 months, 37 to 72 months, 73 months or older) groups. If the patient had multiple visits, body weight and reproductive status of the most recent oral procedure were recorded.

### 2.2 Oral pathology

Oral examination charts, dental radiographs, and clinical photographs were evaluated to identify TDI, tooth wear (abrasion and attrition), and periodontal disease. TDI were classified as enamel fracture (EF), enamel-dentin fracture (UCF), enamel-dentin-pulp fracture (CCF), complicated crown-root fracture (CCRF), uncomplicated crown-root fracture (UCRF), root fracture (RF), tooth luxation (TL), and tooth avulsion (TA). Retained tooth roots were considered RF for the purpose of this study. Periodontal disease was assessed only in strategic teeth (canine, maxillary fourth premolar, and mandibular first molar teeth). Staging of periodontal disease was based on clinical evaluation of the furcation involvement and radiographic evaluation of alveolar bone loss according to the AVDC nomenclature (11).

### 2.3 Statistical analysis

Descriptive statistics for categorical variables (TDI type, tooth type, sex, age, breed, periodontal disease stage) were reported as frequency counts and percentages. Inference statistical analysis was conducted in three steps. First, exploratory analysis based on Spearman’s rank correlation was conducted to identify independent variables that show a statistical trend (*p* < 0.2) of association with the outcome of interest. Second, a purposeful stepwise backwards algorithm was used to identify a subset of independent variables that are associated with the outcome. Third, a multivariable regression analysis was performed to assess the association between outcomes and independent variables of the model. Independent categorical variables assessed were working dog status, tooth type, TDI type, sex, age, body weight, jaw location, and strategic teeth status. The associations were reported either as odds ratio (OR) for binary and ordered categorical outcomes, incidence rate ratio (IRR) for count outcomes, or relative risk ratio (RRR) for unordered categorical outcomes, with their respective 95% CI and *p*-values. All analyses were conducted with Stata 18MP, StataCorp, College Station, TX, with two-sided tests of hypotheses and a *p* < 0.05 as the criterion for statistical significance.

## 3 Results

### 3.1 Patient demographics

The same number of dogs (*n* = 51) were included in both groups. In the WD group, male dogs were overrepresented (*n* = 41; 80.4%), with 19 (46.3%) being neutered and 22 (53.7%) intact. There were 10 (19.6%) female dogs, of which eight (80%) were spayed and two (20%) were intact. The mean age was 70.3 ± 30.8 months and the mean body weight 32.9 ± 6.9 kg in the WD group. Dog breeds included German shepherd (*n* = 22; 43.1%), Belgian malinois (*n* = 14; 27.5%), mixed breed (*n* = 5; 9.8%), German shorthaired pointer (*n* = 4; 7.8%), Labrador retriever (*n* = 3; 5.8%), and Dutch shepherd (*n* = 3; 5.8%).

In the NWD group, there were 29 (56.9%) male dogs of which 23 (79.3%) were neutered and six (20.1%) intact, and 22 (43.1%) female dogs of which 21 (95.5%) were spayed and one (4.55%) intact. The mean age was 104.3 ± 40.3 months, and the mean body weight was 13.0 ± 11.4 kg. The most common breeds were mixed breed (16; 31.4%), Chihuahua (4; 7.8%), Yorkshire terrier (3; 5.9%), and Maltese (3; 5.9%). There were two dogs (3.9%) of each of the following breeds: pug, Portuguese water dog, beagle, and Havanese, and one dog (1.9%) belonging to 17 other breeds.

### 3.2 Oral pathology

#### 3.2.1 Traumatic dentoalveolar injury (TDI)

**Figure 1:**
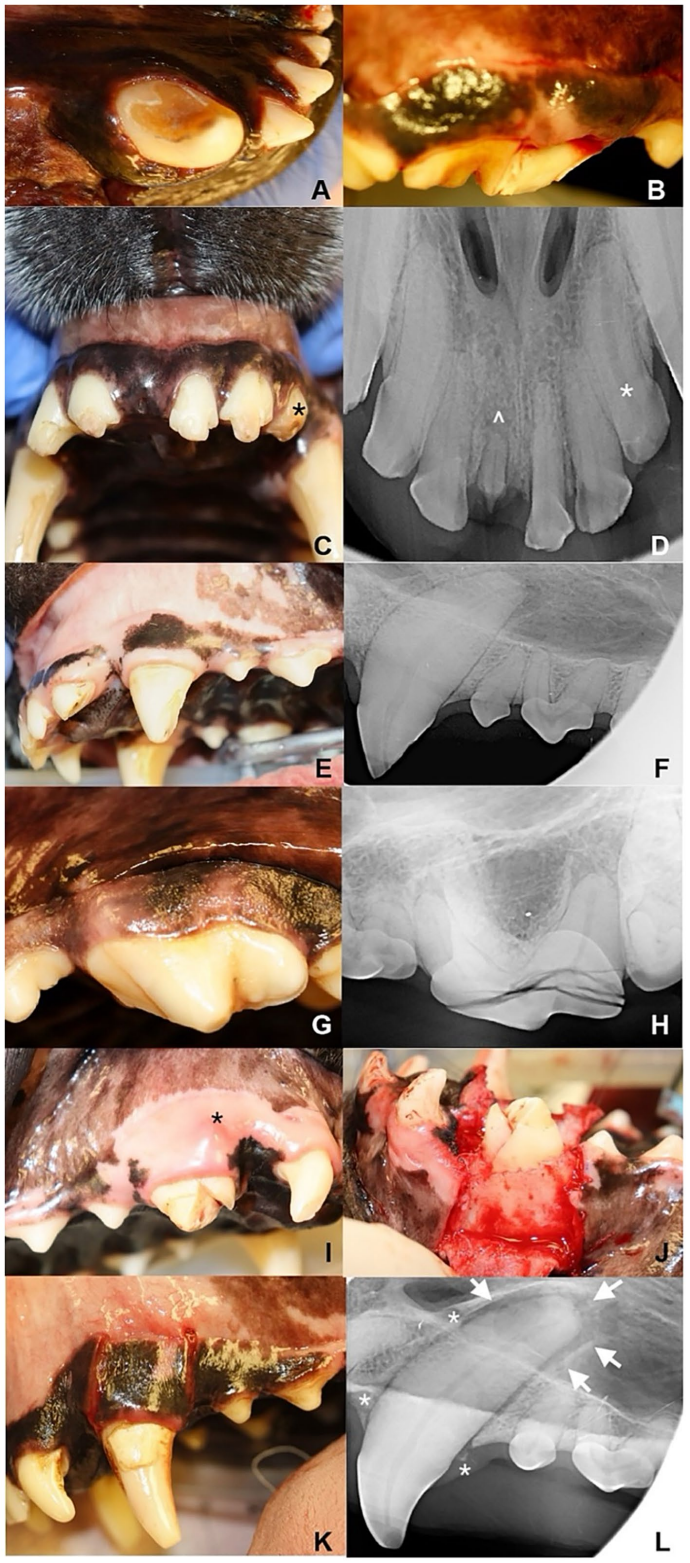
Traumatic dentoalveolar injuries. **(A)** Enamel fracture of the distal aspect of the right mandibular canine tooth. The edges of the enamel are irregular. **(B)** Uncomplicated crown-root fracture of the right maxillary fourth premolar tooth. Mesiobuccal part of the crown is missing without pulp exposure. The fracture extends subgingivally into the root. **(C,D)** Enamel-dentin fracture (UCF), enamel-dentin-pulp fracture (CCF), and root fracture (RF). UCFs present in right maxillary second and third incisor teeth (102, 103) and left maxillary first and second incisor teeth (201, 202). Part of the crown is missing and the surface is irregular. Right maxillary first incisor tooth (101) is missing **(C)**. The retained root (^) of right maxillary first incisor tooth (RF) is shorter than the left maxillary first incisor tooth (201) and shows signs of extrusion **(D)**. About half of the crown (*) of the left maxillary third incisor tooth (203) is missing causing pulp exposure **(C)**. Relative enlarged pulp canal (*) of left maxillary third incisor tooth is visible on **(D)** secondary to CCF. **(E,F)** Enamel-dentin-pulp fracture (CCF) of the left maxillary canine tooth (204). A black dot is visible in the distal surface of the tooth **(E)**. Part of the crown is missing, there is no enamel on the distal aspect of the crown in the fracture site and a periapical lucency **(F)**. **(G,H)** Complicated crown-root fracture (CCRF) of the left maxillary fourth premolar tooth (208), the slab fracture is visible in **(G)**. The dental radiograph shows multiple fracture lines in the crown with a few lines extending into the distal root of left maxillary fourth premolar tooth (208) **(H)**. **(I,J)** Complicated crown-root fracture (CCRF) of the right maxillary canine tooth (104). Inflamed area in the attached gingiva (*) shows the apical extension of the fragments **(I)**. A gingival flap and alveoloplasty are usually necessary to explore the fracture, remove tooth fragments and decide whether the tooth is a candidate for an apical positioning flap **(J)**. **(K,L)** Tooth luxation of the left maxillary canine tooth (204). Mesial and distal gingival lacerations are common secondary to the tooth displacement and fracture of the alveolar bone on lateral luxations **(K)**. The periodontal ligament space is enlarged (arrow). Alveolar fractures are visible (*).

There were a total of 385 TDI for all 102 dogs, with 319 (82.3%) in the WD group and 66 (17.1%) in the NWD group. In the WD group, 48 (94.1%) dogs had at least one TDI, and there was an average of 6.3 ± 6.4 TDI per dog. The most frequent TDI were UCF (*n* = 136; 42.6%), EF (*n* = 79; 24.8%), CCF (*n* = 46; 14.4%), CCRF (*n* = 31; 9.7%), and RF (*n* = 21; 6.6%) (Table 1). In the NWD group, 31 (60.8%) dogs had at least one TDI and an average of 1.3 ± 1.6 TDI per dog. The most frequent TDI were UCF (*n* = 29; 43.9%), RF (*n* = 12; 18.2%), CCF (*n* = 9; 13.6%), EF (*n* = 9; 13.6%), and CCRF (*n* = 5; 7.6%) (Table 1) (Figure 1). UCRF and tooth luxation were the least common TDI in both groups. Most TDI occurred in the upper jaw in both groups (WD 182 [57.1%]; NWD 46 [69.7%]), in incisor teeth in WD (157; 49.2%) and premolar teeth in NWD (31; 47%) (Tables 2, 3). The heaviest weight (>20 kg) and oldest age (≥73 months) groups revealed the most TDI in both WD and NWD (Table 4).

**Table 1:** Traumatic dentoalveolar injury (TDI) in working (WD) and non-working (NWD) dogs.

TDI	Overall, *N* (%)	WD, *N* (%)	NWD, *N* (%)
EF	88 (22.8%)	79 (24.8%)	9 (13.6%)
UCF	165 (42.8%)	136 (42.6%)	29 (43.9%)
UCRF	5 (1.3%)	4 (1.3%)	1 (1.5%)
CCF	55 (14.3%)	46 (14.4%)	9 (13.6%)
CCRF	36 (9.4%)	31 (9.7%)	5 (7.6%)
RF	33 (8.6%)	21 (6.6%)	12 (18.3%)
Lux	3 (0.8%)	2 (0.6%)	1 (1.5%)
Total	385 (100%)	319 (100%)	66 (100%)

TDI, traumatic dentoalveolar injury; WD, working dog; NWD, non-working dog; EF, enamel fracture; UCF, uncomplicated crown fracture (enamel-dentin fracture); UCRF, uncomplicated crown-root fracture; CCF, complicated crown fracture (enamel-dentin-pulp fracture); CCRF, complicated crown-root fracture; RF, root fracture; Lux, tooth luxation.

**Table 2:** Traumatic dentoalveolar injury (TDI) distribution in relation to location and tooth type in working dogs (WD).

Jaw location	Tooth type	EF, *N* (%)	UCF, *N* (%)	UCRF, *N* (%)	CCF, *N* (%)	CCRF, *N* (%)	RF, *N* (%)	Lux, *N* (%)	Total
Upper jaw	I	23 (25.3%)	36 (39.5%)	1 (1.1%)	16 (17.6%)	10 (11%)	4 (4.4%)	1 (1.1%)	91 (100%)
	C	8 (22.2%)	9 (25%)	0	12 (33.3%)	5 (13.9%)	1 (2.8%)	1 (2.8%)	36 (100%)
	PM	14 (26.9%)	29 (55.8%)	1 (1.9%)	0	2 (3.9%)	6 (11.5%)	0	52 (100%)
	M	2 (66.7%)	0	0	0	1 (33.3%)	0	0	3 (100%)
	Total	47 (25.8%)	74 (40.7%)	2 (1.1%)	28 (15.4%)	18 (9.9%)	11 (6%)	2 (1.1%)	182 (100%)
Lower jaw	I	6 (9.1%)	35 (53.1%)	2 (3%)	8 (12.1%)	8 (12.1%)	7 (10.6%)	0	66 (100%)
	C	10 (28.6%)	11 (31.4%)	0	9 (25.7%)	5 (14.3%)	0	0	35 (100%)
	PM	8 (33.3%)	12 (50%)	0	1 (4.2%)	0	3 (12.5%)	0	24 (100%)
	M	8 (66.7%)	4 (33.3%)	0	0	0	0	0	12 (100%)
	Total	32 (23.4%)	62 (45.2%)	2 (1.5%)	18 (13.1%)	13 (9.5%)	10 (7.3%)	0	137 (100%)

*N*, number of teeth affected; I, incisor teeth; C, canine teeth; PM, premolar teeth; M, molar teeth; EF, enamel fracture; UCF, uncomplicated crown fracture (enamel-dentin fracture); UCRF, uncomplicated crown-root fracture; CCF, complicated crown fracture (enamel-dentin-pulp fracture); CCRF, complicated crown-root fracture; RF, root fracture; Lux, tooth luxation.

**Table 3:** Traumatic dentoalveolar injury (TDI) distribution in relation to location and tooth type in non-working dogs (NWD).

Jaw location	Tooth type	EF, *N* (%)	UCF, *N* (%)	UCRF, *N* (%)	CCF, *N* (%)	CCRF, *N* (%)	RF, *N* (%)	Lux, *N* (%)	Total
Upper jaw	I	3 (16.7%)	9 (50%)	0	3 (16.7%)	0	3 (16.6%)	0	18 (100%)
	C	1 (16.7%)	2 (33.3%)	0	1 (16.7%)	1 (16.6%)	0	1 (16.7%)	6 (100%)
	PM	1 (5.3%)	12 (63.1%)	0	1 (5.3%)	4 (21.1%)	1 (5.2%)	0	19 (100%)
	M	0	0	1 (33.3%)	0	0	2 (66.7%)	0	3 (100%)
	Total	5 (10.8%)	23 (50%)	1 (2.2%)	5 (10.9%)	5 (10.9%)	6 (13%)	1 (2.2%)	46 (100%)
Lower jaw	I	1 (25%)	1 (25%)	0	2 (50%)	0	0	0	4 (100%)
	C	2 (66.7%)	0	0	1 (33.3%)	0	0	0	3 (100%)
	PM	1 (8.3%)	4 (33.4%)	0	1 (8.3%)	0	6 (50.0%)	0	12 (100%)
	M	0	1 (100%)	0	0	0	0	0	1 (100%)
	Total	4 (20%)	6 (30%)	0	4 (20%)	0	6 (30%)	0	20 (100%)

*N*, number of teeth affected; I, incisor teeth; C, canine teeth; PM, premolar teeth; M, molar teeth; EF, enamel fracture; UCF, uncomplicated crown fracture (enamel-dentin fracture); UCRF, uncomplicated crown-root fracture; CCF, complicated crown fracture (enamel-dentin-pulp fracture); CCRF, complicated crown-root fracture; RF, root fracture; Lux, tooth luxation.

**Table 4:** Traumatic dentoalveolar injury (TDI) in relation to body weight and age.

	All dogs	WD	NWD		All dogs	WD	NWD
Body weight	TDI count	TDI count	TDI count	Age	TDI count	TDI count	TDI count
<10 kg	25	0	25	≤36 months	34	33	1
10–20 kg	12	1	11	37–72 months	134	121	13
>20 kg	348	318	30	≥73 months	217	165	52

TDI, traumatic dentoalveolar injury; WD, working dog; NWD, non-working dog.

Being a WD was significantly associated with TDI count. When adjusted for age, body weight, sex, and reproductive status, being a WD had a 4-fold increase of having a higher number of TDI than being a NWD (IRR = 3.73; *p* < 0.001; 95% Cl 2.61, 5.33) (Supplementary Table S1). When adjusted for age, body weight, sex, and reproductive status, older dogs (≥73 months) had a 59% increased risk of having a higher number of TDI (IRR = 1.59; *p* = 0.016; 95% CI: 1.09, 2.33) compared to younger dogs (≤36 months) (Supplementary Table S1). When adjusted for confounders and fixed effects, larger dogs (≥20 kg) had a 2-fold increase in having a higher number of TDI (IRR = 3.05; *p* < 0.001; 95% CI: 1.83, 5.09) compared to smaller dogs (<10 kg) (Supplementary Table S1). Compared to female intact dogs, female spayed dogs had a 6-fold increase of having a higher number of TDI (IRR = 5.91; *p* < 0.001; 95% CI: 2.40, 14.56), and male neutered dogs had a 3.4-fold increase of having a higher number TDI (IRR = 3.45; *p* = 0.01; 95% CI: 1.40, 8.47) (Supplementary Table S1).

No association was found between specific TDI and WD/NWD status. However, CCF were significantly associated with age and tooth type, CCRF with tooth type, UCF with tooth type and being a strategic tooth, and RF with body weight and age. For each month increase in age, the likelihood of having a CCF decreased by 1% (OR = 0.99; *p* = 0.01; 95% CI: 0.98, 0.99). Compared to canine teeth, incisor teeth had a 58% reduction in the likelihood of having a CCF (OR = 0.42; *p* = 0.00; 95% CI: 0.23, 0.76), molar teeth had a 98% reduction in the likelihood of having a CCF (OR = 0.02; *p* < 0.001; 95% CI: 0.00, 0.34), and premolar teeth had a 94% reduction in the likelihood of having a CCF (OR = 0.06; *p* < 0.001; 95% CI: 0.02, 0.20) (Supplementary Table S2).

Compared to canine teeth, molar teeth had a 86% reduction in likelihood of having a CCRF (OR = 0.14; *p* = 0.03; 95% CI: 0.03, 0.79), and premolar teeth had a 72% reduction in the likelihood of having a CCRF (OR = 0.28; *p* = 0.01; 95% CI: 0.10, 0.74) (Supplementary Table S2). Compared to canine teeth, the likelihood of having a UCF was 9-fold higher for incisor teeth (OR = 9.6; *p* < 0.001; 95% CI: 4.13, 22.29) and 6-fold higher for premolar teeth (OR = 6.01; *p* < 0.001; 95% CI: 2.88, 12.51). Relative to non-strategic teeth, strategic teeth had a 5-fold increase in likelihood of having a UCF (OR = 5.78; *p* < 0.001; 95% CI: 2.98, 11.19) (Supplementary Table S2). When adjusted for age and strategic tooth classification, for every kilogram increase in body weight, there was a 4% reduction of likelihood of sustaining a RF (OR = 0.96; *p* = 0.03; 95% CI: 0.92, 0.99). When adjusted for body weight and strategic tooth classification, for every month increase in age, there was a 2% increase in likelihood of sustaining a RF (OR = 1.02; *p* < 0.001; 95% CI: 1.01, 1.03). There was an 88% reduction in the likelihood of sustaining a RF in a strategic tooth (OR = 0.12; *p* = 0.01; 95% CI: 0.02, 0.61) (Supplementary Table S2).

#### 3.2.2 Tooth wear

**Figure 2:**
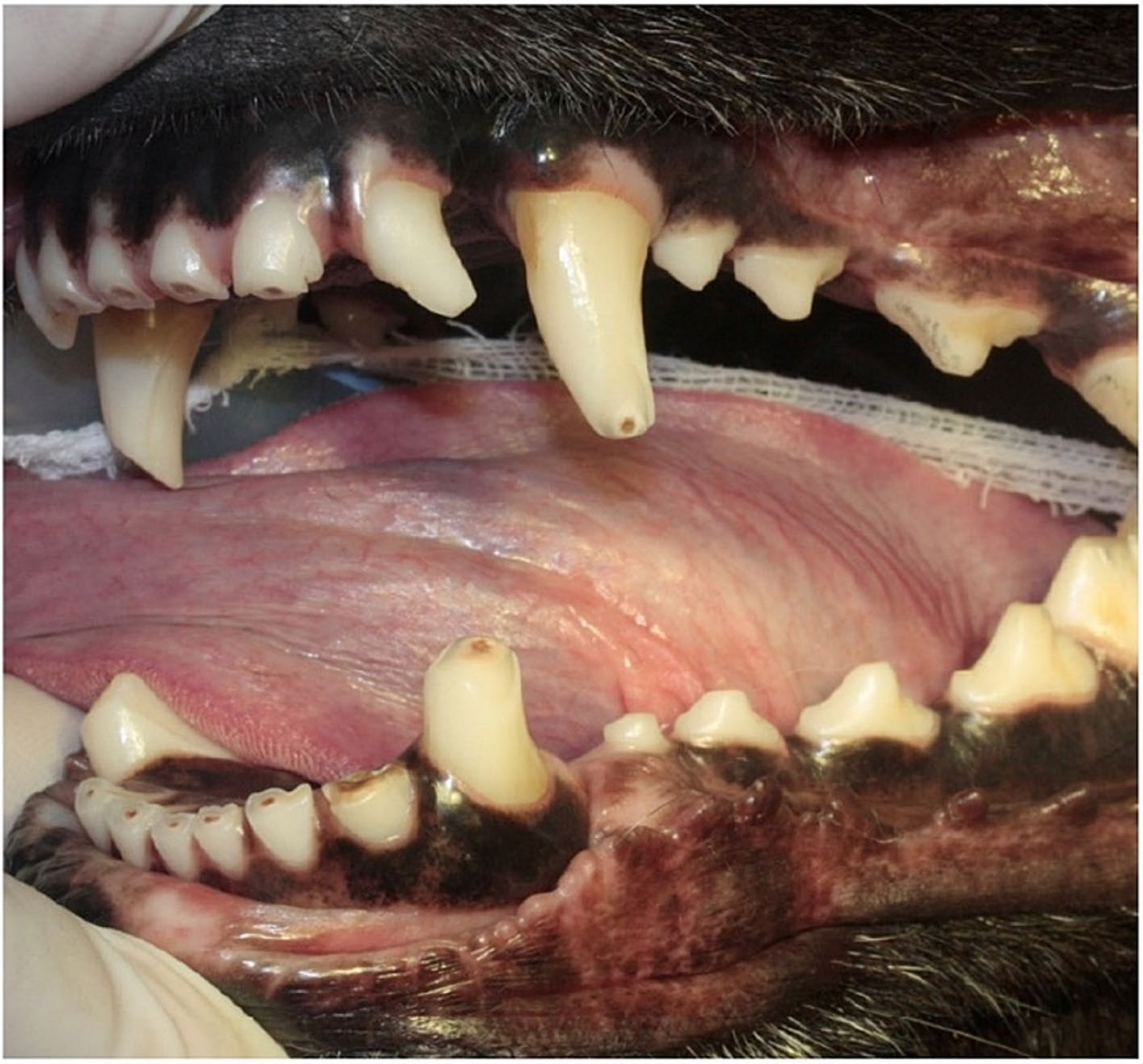
Tooth wear. Severe case of tooth wear secondary to bite work in a working dog. Maxillary and mandibular incisor and canine teeth have lost part of the enamel and dentin. Tertiary dentin is visible in the center of the dental flattened surface. The cusps of the premolar teeth are also flattened.

Tooth wear was identified at least in one tooth in 46 (90.2%) WD and 17 (33.3%) NWD. The mean number of teeth affected was 12.7 ± 11.5 in WD and 1.9 ± 4.9 in NWD, and the difference between both groups was statistically significant (*p* = 0.008; Figure 2). Tooth wear was mostly present in incisor teeth in both groups, with 37.9% (*n* = 245) in WD and 35% (*n* = 35) in NWD (Table 5). Premolar teeth were the second most common tooth type with tooth wear in both groups, with 30.9% (*n* = 200) in WD and 30% (*n* = 30) in NWD (Table 5). Relative to NWD, WD have a 66% increase in likelihood of having tooth wear (OR = 1.66; *p* = 0.01; 95% CI: 1.14, 2.41). Relative to canine teeth, incisor teeth have a 52% reduction in likelihood of having tooth wear (OR = 0.48; *p* = 0.03, 95% CI: 0.24, 0.94). When adjusted for working dog status, sex, tooth type, relative to mandibular teeth, maxillary teeth had a 35% decrease in likelihood of having tooth wear (OR = 0.65; *p* < 0.001; 95% CI: 0.48, 0.86). Relative to non-strategic teeth, strategic teeth had a 41% decrease in likelihood of having tooth wear (OR = 0.59; *p* = 0.05; 95% CI: 0.35, 1.01; Supplementary Table S3).

**Table 5:** Tooth wear (abrasion/attrition) frequency in working dogs (WD) and non-working dogs (NWD).

Working dog status	I, *N* (%)	Max C, *N* (%)	Man C, *N* (%)	PM (non-max PM4), *N* (%)	PM4, *N* (%)	M (non-man M1), *N* (%)	M1, *N* (%)	Total, *N* (%)
WD	245 (37.9%)	47 (7.3%)	48 (7.4%)	178 (27.6%)	22 (3.4%)	71 (11%)	35 (5.4%)	646 (100%)
NWD	35 (35%)	12 (12%)	12 (12%)	28 (28%)	2 (2%)	3 (3%)	8 (8%)	100 (100%)

WD, working dog; NWD, non-working dog; I, incisor teeth; Max C, maxillary canine teeth; Man C, mandibular canine teeth; PM, premolar teeth; max PM4, maxillary fourth premolar teeth; M, molar; man M1, mandibular first molar teeth.

#### 3.2.3 Periodontal disease

A total of 696 strategic teeth (canine, maxillary fourth premolar, mandibular first molar) were evaluated for periodontal disease between the two groups (308 teeth in WD and 388 teeth in NWD). All potential strategic teeth were not evaluated in each dog due to some dogs in the WD group had only fully evaluated the primary complaint (ex. fractured tooth) and dogs in the NWD group lost strategic teeth prior to our evaluation. In the WD group, there were 223 (72.4%) strategic teeth with stage 1, 79 (25.6%) teeth with stage 2, 2 (0.7%) teeth with stage 3, and 4 (1.3%) teeth with stage 4 periodontal disease. In the NWD group, there were 235 (60.6%) strategic teeth with stage 1, 78 (20.1%) teeth with stage 2, 35 (9.0%) teeth with stage 3, and 40 (10.3%) teeth with stage 4 periodontal disease (Table 6).

**Table 6:** Periodontal disease stages of strategic teeth in 51 working dogs (WD) and 51 non-working dogs (NWD).

Strategic tooth type	Stage 1, *N* (%)		Stage 2, *N* (%)		Stage 3, *N* (%)		Stage 4, *N* (%)	
	WD	NWD	WD	NWD	WD	NWD	WD	NWD
Max C	25 (11.3%)	41 (17.5%)	57 (72.1%)	40 (51.3%)	0	10 (28.6%)	2 (50.0%)	9 (22.5%)
Man C	69 (30.9%)	65 (27.7%)	13 (16.5%)	15 (19.2%)	0	9 (25.7%)	0	12 (30%)
Max PM4	60 (26.9%)	61 (26%)	7 (8.9%)	7 (9%)	2 (100%)	9 (25.7%)	2 (50.0%)	13 (32.5%)
Man M1	69 (30.9%)	68 (28.8%)	2 (2.5%)	16 (20.5%)	0	7 (20%)	0	6 (15%)
Total	223 (100%)	235 (100%)	79 (100%)	78 (100%)	2 (100%)	35 (100%)	4 (100%)	40 (100%)

*N*, number of teeth affected; WD, working dog; NWD, non-working dog; Max C, maxillary canine teeth; Man C, mandibular canine teeth; Max PM4, maxillary fourth premolar teeth; Man M1, mandibular first molar teeth.

The analysis revealed that sex and reproductive status were not associated with periodontal disease. Analysis was adjusted for WD/NWD status and for age, sex, and body weight. In comparison to small dogs (≤10 kg), medium-sized (10–20 kg) and large dogs (>20 kg) were 84% (OR 0.16; *p* = 0.04; 95% CI: 0.03, 0.89) and 96% (OR 0.04; *p* = 0.00; 95% CI: 0.01, 0.22) less likely to have advanced stages of periodontal disease, respectively. In comparison to ≤36 month-old dogs, ≥73 month-old dogs were 13-fold more likely to have severe stages of periodontal disease (OR 12.99; *p* = 0.002; 95% CI: 2.51, 67.36). In comparison to mandibular teeth, maxillary teeth were 7-fold more likely to have more advanced stages of periodontal disease (OR 6.9; *p* < 0.001; 95% CI: 3.72, 12.83) (Supplementary Table S4).

When base category was periodontal disease stage 1 and when adjusted for WD/NWD status, sex, and body weight, for every 1 month increase in age, the likelihood of having stage 2 periodontal disease increases by 1% (RRR = 1.01; *p* = 0.001; 95% CI: 1.00, 1.01) (Supplementary Table S5). When adjusted for WD/NWD status, sex, and age, for every 1 kg increase in body weight, the likelihood of having stage 2 periodontal disease increases by 4% (RRR = 0.96; *p* = 0.001; 95% CI: 0.94, 0.98) (Supplementary Table S5).

## 4 Discussion

This study evaluated the prevalence of TDI, tooth wear, and periodontal disease in working dogs (WD) and non-working dogs (NWD). The prevalence of TDI was 94.1% in WD and 60.8% NWD, which is higher than previously reported (26.2–43.6%) (7, 8). This difference may be because different types of TDI and other inclusion criteria were used in the previous studies. Less severe trauma for which treatment may not have been necessary at presentation was assessed in the present study, while a previous study utilized electronic medical records which did not contain all oral examination findings (7). That study also included additional TDI such as enamel infraction, concussion, subluxation, avulsion, and alveolar fracture, which were not noted in the present study.

Another study found the prevalence of TDI in military working dogs to be higher (43.6%) than in pet dogs and cats; however, that study focused mainly on UCF (enamel-dentin) and CCF (enamel-dentin-pulp fracture) (8). In the present study, WD had a 4-fold increase in TDI compared to NWD. WD are more exposed to various sources of trauma during training and work. They may also be more prone to TDI because they are physically active and may live in different housing environments (kennels). Based on the results of the present study, frequent oral examinations are justified to identify and treat TDI and take preventive measures such as behavior modification, and changes in training and reward strategies. As previously reported (8), older dogs had more TDI than younger dogs due to the fact of having more opportunities to sustain trauma over their lifetime. Many TDI are diagnosed incidentally during an anesthetized oral examination, without knowledge of a specific date of the injury. In addition, breed, size, skull shape, and body size affect bite forces (12). Most dogs in the WD group in the present study belonged to specific breeds and were larger than in the NWD group.

The most common tooth type affected with a TDI in the present study were incisor teeth in WD and premolar teeth (including the maxillary fourth premolar teeth) in NWD. Both tooth types together were most frequently affected in each group, with canine teeth ranking third in place. The higher prevalence of TDI affecting incisor teeth in WD may be due to the high-impact nature of their work, including bite work, scent work, and handling heavy objects, and their position at the front of the mouth make them more vulnerable with certain activities to direct traumatic impact. On the contrary, premolar teeth of NWD may sustain more TDI due to improper chewing behavior. The cause of TDI was not determined in the present study, but many TDI were incidental findings and located in premolar teeth, whose main function is chewing. The occurrence of TDI may also be influenced by routine and repetitive activities such as playing with hard objects (sticks, rocks, toys). The military WD study found housing environment the second most common cause of dental trauma (8). The same study also found that there was no statistical significance between certification category and incidence of TDI with patrol functions. In the present study, TDI cause and type of work were not able to be recorded for most cases included.

Being a WD was only a risk factor for the overall TDI count but not for specific types of TDI. The most common TDI was an uncomplicated crown fracture (UCF) for both groups, followed by root fracture (RF) in NWD and enamel fracture (EF) in WD. In previous studies, RF have been associated with incisor teeth and occurring more likely in the lower than upper jaw (7). The present study did not find an association with tooth type, but RF was more likely to occur in smaller and older dogs. The dogs included in the NWD group were smaller and older than the dogs included in the WD group. Attachment loss secondary to periodontal disease and other characteristics of small breed dogs may potentially affect tooth resistance to trauma.

Compared to canine teeth, premolar and molar (including carnassial) teeth were less likely to sustain a CCF or CCRF. Fracture resistance depends on crown height and diameter, amount of dentin, and direction of forces applied to a tooth (13–15). The crowns of canine teeth are longer than in other teeth, and they sustain trauma from different directions, disto-mesial during bite work and mesio-distal during blunt trauma (hits, falls, and other impacts against a hard surface).

Strategic teeth had a higher risk of UCF than non-strategic teeth, and incisor and premolar teeth had a higher risk of UCF than canine teeth. Maxillary fourth premolar teeth belong to both groups (strategic and premolar teeth) and are functionally most important teeth. Although studies evaluated the forces at predetermined directions upon which canine and maxillary fourth premolar teeth fracture, it is unknown which trauma (magnitude and direction) will cause uncomplicated versus complicated tooth fractures (14, 15). A study looking at UCF in maxillary fourth premolar teeth found that 1 out of 4 teeth showed radiographic lesions of endodontic origin despite not having clinical pulp exposure, and larger dogs had a higher risk of having a UCF alone and UCF with periapical pathology (16).

Tooth wear in this study included abrasion and attrition. Tooth wear was mostly present in incisor teeth in both WD and NWD groups, which is a similar finding in a Brazilian study (17). Some mild class 2 and 3 malocclusions can cause attrition of incisor and canine teeth. Many WD had metal staining and distal abrasion of canine teeth. Hyperactivity in WD that use an abrasive object such as a metal bowl or cage bar to chew on may cause tooth abrasion (18). There is lack of veterinary literature in tooth wear and its consequences. Abrasion and attrition are a form of trauma that cause concussive injury of the tooth, with gradual loss of tooth structure, exposure of dentinal tubules, and deposition of tertiary dentin. The microleakage of bacteria through dentinal tubules can result in pulpal infection and/or root resorption (19). Tooth vitality may be compromised, and chronic inflammation may also lead to pulp necrosis and radicular cyst formation (20).

Most strategic teeth (canine, maxillary fourth premolar, mandibular first molar teeth) in WD of the present study were classified as stage 1 or 2 periodontal disease, while in NWD there was more variability with higher numbers classified as stage 3 and 4 periodontal disease. Although periodontal disease occurs in dogs of all ages and sizes, small breed dogs (<6.5 kg) are five times more likely to be diagnosed with periodontal disease than large breed dogs (>25 kg). In addition, the disease is chronic and progressive; therefore, the prevalence is higher in older dogs (6). Being a WD does not seem to influence the severity of periodontal disease in strategic teeth, and the lower prevalence of strategic teeth with stages 3 and 4 periodontal disease in WD may more likely be related to dog size and breed than WD/NWD status.

WD had no mandibular first molar teeth with stage 3 or 4 periodontal disease. A previous study showed small dogs to have proportionally larger mandibular first molar teeth relative to mandibular height compared to larger breed dogs, which may contribute to increased susceptibility for periodontitis and tooth loss in small dogs (21). Another study also showed significantly thinner gingiva and alveolar bone in toy breed dogs compared to small and medium breed dogs, which may be a contributing factor for a higher prevalence of periodontitis in smaller dog breeds (22).

Dogs and cats with periodontal disease may have reduced biting abilities due to oral pain. The loading force during mastication is generated by the masticatory muscles. Mechanoreceptors in the periodontal ligament respond to the force applied to the tooth crown. If there is decreased periodontal health, this could reduce bite force (12). The present study showed that NWD have higher prevalence of stage 3 and 4 periodontal disease in strategic teeth, which may be explained due to less bite force exerted onto the teeth and thus fewer TDI.

The present study was retrospective in nature. Oral examination charts may not have included all the findings if they were deemed not sufficiently relevant for the purpose of the visit, or they were not recorded because they did not need treatment. Periodontal disease was only evaluated in strategic teeth based on dental radiography and furcation involvement. Some dogs did not have one or more strategic teeth, which may have been lost due to periodontal disease or fractures prior to our evaluations. Staging of periodontal disease based only on dental radiographs and furcation may have not been completely accurate due to the difficulty in evaluating lingual, palatal and vestibular areas. In addition, attachment loss determined by periodontal pocket depth and gingival recession were not considered due to insufficient information in WD. Because strategic teeth have important function in apprehension, the present study assessed only these teeth, as periodontal compromise or loss of them could affect the WD’s career. Another study limitation was patient selection. WD were not matched to NWD with similar ages, body weights, or breeds. Patients usually seen at the authors’ institution are companion animals, which often tend to be small to medium-sized dogs that usually need more dental and periodontal care compared to large dogs. This study design was reasonable to find differences between prevalence of TDI and tooth wear in both groups, however, the statistical power was low to detect statistical differences between the different stages of periodontal disease.

In conclusion, TDI and tooth wear are more common in WD than in NWD, and their prevalence is higher in the present study than has previously been reported, with 9 of 10 WD having at least one TDI and tooth wear. There was no significant difference of specific types of TDI between WD and NWD. Also, there was no significant difference in prevalence of periodontal disease in strategic teeth between WD and NWD. As many injuries are incidental findings, dog handlers/owners and veterinarians should proactively act to prevent, diagnose, and treat them if deemed necessary. Periodontal disease also is common in WD, but it was not severe enough to cause compromise or loss of the tooth. Daily home oral hygiene measures and regular comprehensive oral health assessment and treatment are recommended to maintain good oral health.

## Data Availability

The original contributions presented in the study are included in the article/Supplementary material, further inquiries can be directed to the corresponding author.
