# Supplementary material for: Traumatic dentoalveolar injury, tooth wear, and periodontal disease in working and non-working dogs (2018–2022)
Source: Front Vet Sci. 2026 May 7;13:1816961. doi: 10.3389/fvets.2026.1816961 (PMC13189841; doi:10.3389/fvets.2026.1816961)
Supplement: Supplementary file 1 [file Table_1.docx]

**SUPPLEMENTARY MATERIAL**

**Table S1. Poisson Regression Analysis of TDI count**

| **TDI count** | **IRR** | **P > \|z\|** | **95% confidence interval** | |
| --- | --- | --- | --- | --- |
| **Non-working dogs** | Reference category |  |  | |
| **Working** | 3.73 | 0.00 | 2.61 | 5.33 |
|  |  |  |  | |
| **Age ≤ 36 months** | Reference category |  |  |  |
| **Age 37-72 months** | 1.18 | 0.39 | 0.81 | 1.73 |
| **Age ≥ 73 months** | 1.59 | 0.02 | 1.09 | 2.33 |
|  |  |  |  |  |
| **Weight <10kg** | Reference category |  |  |  |
| **Weight 10-20kg** | 1.25 | 0.54 | 0.62 | 2.52 |
| **Weight >20kg** | 3.05 | 0.00 | 1.83 | 5.09 |
|  |  |  |  |  |
| **Sex, reproductive status** |  |  |  |  |
| **Female intact** | Reference category |  |  |  |
| **Female spayed** | 5.91 | 0.00 | 2.40 | 14.56 |
| **Male intact** | 1.78 | 0.22 | 0.71 | 4.43 |
| **Male Neutered** | 3.45 | 0.01 | 1.40 | 8.47 |
|  |  |  |  |  |
| **Constant** | 0.14 | 0.00 | 0.05 | 0.39 |

IRR: Incidence rate ratio

**Table S2.**

**Logistic Regression Model of Complicated Crown Fracture**

| **Complicated crown fracture** | **OR** | **P > \|z\|** | **95% confidence interval** | |
| --- | --- | --- | --- | --- |
| **age** | 0.99 | 0.01 | 0.98 | 0.99 |
|  |  |  |  |  |
| **Tooth type:** |  |  |  |  |
| **Canine** | Reference category |  |  |  |
| **Incisor** | 0.42 | 0.00 | 0.23 | 0.76 |
| **Molar** | 0.20 | 0.00 | 0.00 | 0.34 |
| **Pre-molar** | 0.65 | 0.00 | 0.02 | 0.20 |
| **Constant** | 0.40 | 0.02 | 0.18 | 0.87 |

OR: Odds ratio

**Logistic Regression Model of Complicated Crown Root Fracture**

| **Complicated crown root fracture** | **OR** | **P > \|z\|** | **95% confidence interval** | |
| --- | --- | --- | --- | --- |
| **Tooth type:** |  |  |  |  |
| **Canine** | Reference category |  |  |  |
| **Incisor** | 0.63 | 0.24 | 0.29 | 1.35 |
| **Molar** | 0.14 | 0.03 | 0.03 | 0.79 |
| **Pre-molar** | 0.28 | 0.01 | 0.10 | 0.74 |
| **Constant** | 0.08 | 0.00 | 0.04 | 0.14 |

OR: Odds ratio

**Logistic Regression Model of Uncomplicated Crown Fracture**

| **Uncomplicated crown fracture** | **OR** | **P > \|z\|** | **95% confidence interval** | |
| --- | --- | --- | --- | --- |
| **Tooth type:** |  |  |  |  |
| **Canine** | Reference category |  |  |  |
| **Incisor** | 9.60 | 0.00 | 4.13 | 22.29 |
| **Molar** | 0.54 | 0.23 | 0.19 | 1.47 |
| **Pre-molar** | 6.00 | 0.00 | 2.89 | 12.5 |
|  |  |  |  |  |
| **Non-strategic** | Reference category |  |  |  |
| **Strategic** | 5.78 | 0.00 | 2.98 | 11.19 |
|  |  |  |  |  |
| **Sex:** |  |  |  |  |
| **FI** | Reference category |  |  |  |
| **FS** | 5.86 | 0.00 | 2.30 | 14.93 |
| **MC** | 1.58 | 0.34 | 0.62 | 4.02 |
| **MI** | 1.91 | 0.18 | 0.73 | 4.99 |
|  |  |  |  |  |
| **Constant** | 0.01 | 0.00 | 0.00 | 0.04 |

OR: Odds ratio

**Logistic Regression Model of Root Fracture**

| **Root Fracture** | **OR** | **P > \|z\|** | **95% confidence interval** | |
| --- | --- | --- | --- | --- |
| **Weight (kg)** | 0.96 | 0.03 | 0.92 | 0.99 |
| **Age** | 1.02 | 0.00 | 1.01 | 1.03 |
|  |  |  |  |  |
| **Non-strategic** | Reference category |  |  |  |
| **Strategic** | 0.12 | 0.01 | 0.02 | 0.61 |
| **Constant** | 0.04 | 0.00 | 0.01 | 0.23 |

OR: Odds ratio

**Table S3. Firth Logistic Regression Analysis of Tooth wear**

| **Tooth wear** | **OR** | **P > \|z\|** | **95% confidence interval** | |
| --- | --- | --- | --- | --- |
| **Non-working dogs** | Reference category |  |  |  |
| **Working** | 1.66 | 0.00 | 1.14 | 2.41 |
|  |  |  |  |  |
| **Tooth type:** |  |  |  |  |
| **Canine** | Reference category |  |  |  |
| **Incisor** | 0.48 | 0.03 | 0.24 | 0.94 |
| **Molar** | 1.16 | 0.65 | 0.60 | 2.23 |
| **Pre-molar** | 0.64 | 0.15 | 0.35 | 1.18 |
|  |  |  |  |  |
| **Mandible** | Reference category |  |  |  |
| **Maxilla** | 0.65 | 0.00 | 0.48 | 0.86 |
|  |  |  |  |  |
| **Non-strategic** | Reference category |  |  |  |
| **Strategic** | 0.59 | 0.05 | 0.35 | 1.00 |
|  |  |  |  |  |
| **Sex:** |  |  |  |  |
| **Female intact** | Reference category |  |  |  |
| **Female spayed** | 0.38 | 0.01 | 0.19 | 0.79 |
| **Male neutered** | 0.46 | 0.03 | 0.23 | 0.92 |
| **Male intact** | 0.49 | 0.05 | 0.24 | 1.01 |
|  |  |  |  |  |
| **Constant** | 8.47 | 0.00 | 3.19 | 22.42 |

OR: Odds ratio

**Table S4. Ordered Logistic Regression Analysis of Periodontal disease**

| **Periodontal disease** | **OR** | **P > \|z\|** | **95% confidence interval** | |
| --- | --- | --- | --- | --- |
| **Working vs non-working** |  |  |  | |
|  |  |  |  | |
| **Sex** |  |  |  | |
| **Male** | 0.62 | 0.34 | 0.21 | 1.85 |
| **Reproductive status** | 0.45 | 0.19 | 0.14 | 1.48 |
|  |  |  |  | |
| **Weight <10kg** | Reference category |  |  |  |
| **Weight 10-20kg** | 0.16 | 0.04 | 0.03 | 0.89 |
| **Weight >20kg** | 0.04 | 0.00 | 0.01 | 0.22 |
|  |  |  |  | |
| **Age ≤ 36 months** | Reference category |  |  | |
| **Age 37-72 months** | 2.34 | 0.36 | 0.39 | 14.12 |
| **Age ≥ 73 months** | 12.99 | 0.00 | 2.51 | 67.36 |
|  |  |  |  | |
| **Mandible** | Reference category |  |  | |
| **Maxilla** | 6.91 | 0.00 | 3.72 | 12.83 |

OR: Odds Ratio; WD: Working dog; NWD: Non-working dog

**Table S5. Multivariate Logistic Regression Analysis of Periodontal Disease**

| **Periodontal disease** | **RRR** | **P > \|z\|** | **95% confidence interval** | |
| --- | --- | --- | --- | --- |
| **Stage 1** | (base outcome) |  |  |  |
|  |  |  |  |  |
| **Stage 2** |  |  |  |  |
| Non-working | Reference category |  |  |  |
| Working | 2.75 | 0.001 | 1.51 | 5.03 |
| Age | 1.01 | 0.001 | 1.00 | 1.01 |
| Weight (kg) | 0.96 | 0.001 | 0.94 | 0.98 |
|  |  |  |  |  |
| Sex: |  |  |  |  |
|  |  |  |  |  |
| Female spayed | 1.26 | 0.33 | 0.79 | 2.00 |
| Male intact | 1.43 | 0.17 | 0.86 | 2.36 |
|  |  |  |  |  |
| Constant | 0.21 | 0.00 | 0.10 | 0.41 |
|  |  |  |  |  |
| **Stage 3** |  |  |  |  |
| Non-working | Reference category |  |  |  |
| Working | 0.31 | 0.18 | 0.05 | 1.75 |
| Age | 1.03 | 0.00 | 1.02 | 1.04 |
| Weight (kg) | 0.94 | 0.01 | 0.89 | 0.98 |
|  |  |  |  |  |
| Sex |  |  |  |  |
| Female spayed | 6.44 | 0.00 | 2.79 | 14.85 |
| Male intact | 5.49 | 0.01 | 1.45 | 20.75 |
|  |  |  |  |  |
| Constant | 0.00 | 0.00 | 0.00 | 0.027 |
|  |  |  |  |  |
| **Stage 4** |  |  |  |  |
| Non-working | Reference category |  |  |  |
| Working | 10.73 | 0.09 | 0.72 | 160.35 |
| Age | 1.04 | 0.00 | 1.02 | 1.05 |
| Weight (kg) | 0.82 | 0.00 | 0.73 | 0.91 |
|  |  |  |  |  |
| Sex |  |  |  |  |
| Female spayed | 2.01 | 0.14 | 0.79 | 5.12 |
| Male intact | 11.45 | 0.00 | 3.89 | 33.67 |
|  |  |  |  |  |
| Constant | 0.01 | 0.00 | 0.00 | 0.05 |

RRR: Relative risk ratio
